# Bone Marrow Adipocytes: A Critical Player in the Bone Marrow Microenvironment

**DOI:** 10.3389/fcell.2021.770705

**Published:** 2021-11-29

**Authors:** Lipeng Wang, Hao Zhang, Sicheng Wang, Xiao Chen, Jiacan Su

**Affiliations:** ^1^ Institute of Translational Medicine, Shanghai University, Shanghai, China; ^2^ Department of Orthopedics Trauma, Shanghai Changhai Hospital, Naval Medical University, Shanghai, China; ^3^ Department of Orthopedics, Shanghai Zhongye Hospital, Shanghai, China

**Keywords:** bone marrow adipocytes, bone marrow mesenchymal stromal cells, hematopoiesis, osteogenesis, osteoclastogenesis, immune regulation

## Abstract

Recognized for nearly 100 years, bone marrow adipocytes (BMAs) form bone marrow niches that contain hematopoietic and bone cells, the roles of which have long been underestimated. Distinct from canonical white, brown, and beige adipocytes, BMAs derived from bone marrow mesenchymal stromal cells possess unique characteristics and functions. Recent single-cell sequencing studies have revealed the differentiation pathway, and seminal works support the tenet that BMAs are critical regulators in hematopoiesis, osteogenesis, and osteoclastogenesis. In this review, we discuss the origin and differentiation of BMAs, as well as the roles of BMAs in hematopoiesis, osteogenesis, osteoclastogenesis, and immune regulation. Overall, BMAs represent a novel target for bone marrow-related diseases, including osteoporosis and leukemia.

## Introduction

Bone marrow niches are specialized microenvironments that include hematopoietic cells, mesenchymal lineage cells, endothelial cells, and nerves. Erythroid, myeloid, and lymphoid cells constitute the hematopoietic lineage, and mesenchymal stromal cells (MSCs) mainly differentiate into adipocytes, osteoblasts, and chondrocytes (de Paula and Rosen, 2020).

Bone marrow adipocytes (BMAs) were first described in 1922, but it was not until recently that scientists began to understand the basic tenets of BMAs. BMAs are developmentally and functionally distinct from classical white, brown, and beige adipocytes (Sebo et al., 2019). White adipocytes are large, lipid-laden cells that act as an energy source for other tissues and make up 99% of the volume of the subcutaneous and visceral adipose tissue depot. Brown adipocytes are small, mitochondria-abundant cells that burn fatty acids to generate heat. Beige adipocytes reside in subcutaneous depots together with white adipocytes but share some functional features of brown adipocytes, especially in terms of thermogenic capacity through uncoupling protein 1 and morphology (multilocular small cells). BMAs, derived from a unique origin, are metabolically active cells with abundant lipid stores, mitochondria, and endoplasmic reticulum. The amount of BMAs is dynamic during development and in several conditions including osteoporosis, aging, and caloric restriction (de Paula and Rosen, 2020). These observations have encouraged the scientists to explore the roles of BMAs.

## BMA identification

### Origin and classification

Lineage tracing results have indicated that BMAs arise from bone marrow mesenchymal stromal cells (BMSCs), not white adipocytes, brown adipocytes, or hematopoietic progenitors. Efforts are ongoing to identify the adipocyte progenitors *in vivo*. It remains unclear whether BMAs originate from a single population or different sources.

*Lepr* ^+^ BMSCs are a major source of BMAs in adults. BMSCs are heterogeneous and labeled according to the absence of hematopoietic or endothelial markers accompanied by the expression of several stem cell markers (Zhou et al., 2014). In adults, *Lepr* ^*+*^ stromal cells are situated around the vascular network and account for 94% of BMSCs. *Lepr* ^*+*^ cells arise postnatally and form most bone cells and BMAs in adults (Zhou et al., 2014). After irradiation, fracture, or transplantation, *Lepr* ^+^ cells are accountable for the generation of bone and BMAs (Zhou et al., 2014).

Comprising approximately 70% of adult marrow volume and 10% of adipose tissue mass in healthy individuals, bone marrow adipose tissue (BMAT) is mainly formed by BMAs and two distinct compositions exist: constitutive (cBMAT) and regulated (rBMAT) compartments (Scheller et al., 2015). Micro-computed tomography (CT) on osmium tetroxide-stained bone imaging has shown that cBMAT is located in the distal region of the tibia. cBMAT forms immediately after birth and represents a more stable form of BMAT that rarely responds to environmental stimuli. cBMAT development results in an accumulation of cells. rBMAT resides in vertebrae and the proximal region of the tibia and is sensitive to global and local cues, including cold exposure and radiation; it also participates in physiological adaptation. Although the classification of rBMAT and cBMAT is clear within rodents, the extent to which this nomenclature can be applied to humans remains to be determined.

### Differentiation

Although the differentiation routes of BMSCs into adipocytes have been elucidated, the identification and characteristics of the precursors are poorly explored. A recent study depicts the adipose precursor hierarchy of BMSCs (Ambrosi et al., 2017). Focusing on cell surface markers, they showed that CD45^−^CD31^−^Sca^+^CD24^+^ multipotent precursor cells differentiate into both BMAs and osteoblasts. CD45 and CD31 are hematopoietic lineage markers, while Sca and CD24 are adipose precursor markers. This population further differentiates into CD45^−^CD31^−^Sca^+^CD24^−^ adipogenic precursors, which give rise to CD45^−^CD31^−^Sca^-^Zfp234^+^ preadipocytes and then mature BMAs (Grandl and Wolfrum, 2017). Another group identifies new subtypes of *Lepr* ^+^ BMSCs that differentiate into BMAs: *Mpg* ^high^ and *Lpl* ^high^ clusters (Tikhonova et al., 2019).

**FIGURE 1:**
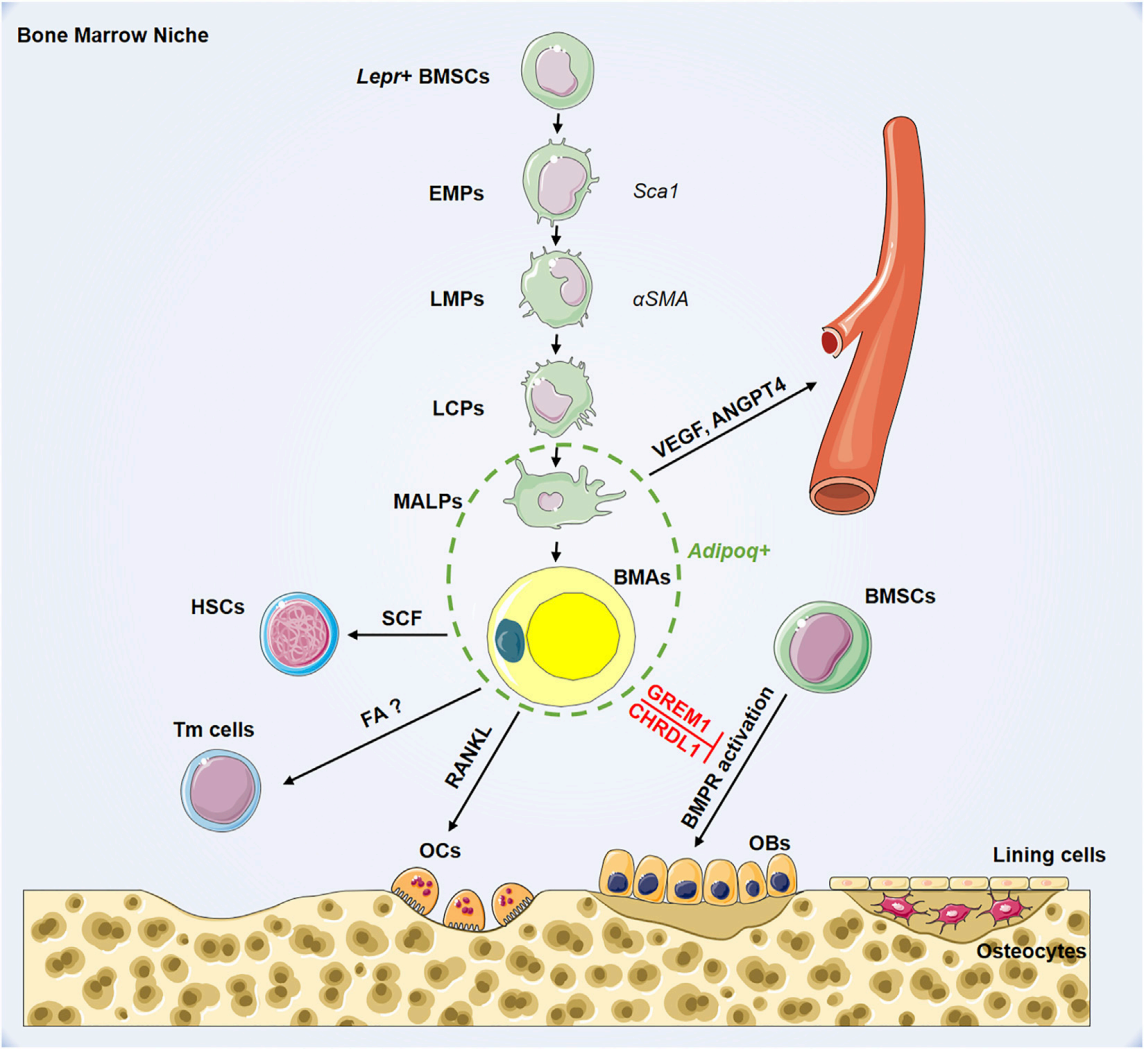
Hierarchy of bone marrow adipocyte lineage cell and its role in bone marrow niche. BMSCs: bone marrow mesenchymal stromal cells; EMPs: early mesenchymal progenitors; LMPs: late mesenchymal progenitors; LCPs: lineage committed progenitors; MALPs: marrow adipogenic lineage precursors; BMAs: bone marrow adipocytes; HSCs: hematopoietic stem cells; Tmcells: T memory cells; OCs: osteoclasts; OBs: osteoblasts; *Lepr*: leptin receptor; *Sca1*: stem cell antigen 1; *αSMA*: smooth muscle actin alpha; *Adipoq*: adiponectin; SCF: stem cell factor; RANKL: receptor activator for nuclear factor-κ B ligand; FAO; VEGF: vascular endothelial growth factor; ANGPT4: angiopoietin 4; GREM1: gremlin1; CHRDL1: chordin-like1; BMPR: bone morphogenetic protein receptor.

To delineate the development of BMSCs into terminal BMAs through hierarchical differentiation paths *in vivo*, Qin et al. performed extensive single-cell RNA-sequencing on BMSCs (Zhong et al., 2020). Mesenchymal lineage cells are divided into nine subpopulations. Analysis of lineage-unique gene markers identified collections of BMAs, osteoblasts, osteocytes, and chondrocytes. Trajectory pattern analysis using the slingshot method identified the most primordial subgroup in the sequencing dataset: early mesenchymal progenitors (EMPs). The results show that EMPs express some stem cell markers, including Sca-1, Thy1, and Cd34. Based on the expression level of osteogenic genes, they also identified intermediate mesenchymal progenitors (IMPs), late mesenchymal progenitors (LMPs), and lineage committed progenitors (LCPs) before differentiation into osteoblasts or BMAs. The authors found that α-smooth muscle actin (SMA) labels mesenchymal progenitors before bifurcated differentiation and can act as a marker for LMPs. Notably, they identified an original adipogenic lineage cell subpopulation, which are lipid-poor, labeled by adiponectin (*Adipoq*)-Cre and can be observed after LCPs and before mature BMAs during adipogenic differentiation. This novel population of adipogenic lineage cells is referred to as marrow adipogenic lineage precursors (MALPs). MALPs maintain marrow vasculature and inhibit bone formation by secreted factors such as vascular endothelial growth factor (VEGF) and angiopoietin 4 (ANGPT4) (Zhong et al., 2020). This study showed relatively comprehensive *in vivo* differentiation of BMSCs into BMAs inside the bone marrow (Figure 1). Overall, there is heterogeneity in adipocytes in bone marrow, and BMAs at different stages of differentiation may play distinct biological roles.

## Roles of BMAs

Adipose tissue is a substantial endocrine organ that regulates physiological activities by secreting hormones and cytokines (Cawthorn et al., 2014; Suchacki et al., 2020). It is predicted that 263 distinct proteins are secreted by primary human adipocytes (Lehr et al., 2012). Although the function of BMAs in marrow niches is under investigation, their regulatory role in hematopoiesis, bone metabolism, and immunity are only partially understood.

### The role of BMAs in hematopoiesis is controversial

Evidence indicates that BMAs negatively regulate hematopoiesis (Naveiras et al., 2009; Scheller et al., 2016). Adipocyte-abundant caudal vertebrae retain fewer hematopoietic stem cells (HSCs) and short-term progenitors compared with adipocyte-free thoracic vertebrae. Furthermore, the genetic and pharmacologic inhibition of adipogenesis accelerates hematopoietic recovery after irradiation and bone marrow transplant (Naveiras et al., 2009). However, it is still unclear if this reflects the influence of BMAs on HSCs or a secondary impact on the marrow environment.

A recent study reported that adipocytes in long bones support hematopoietic restoration following irradiation by providing stem cell factor (SCF), a critical factor for HSC survival (Oguro et al., 2013), whereas in tail vertebrae adipocytes impair hematopoiesis (Zhou et al., 2017). After irradiation, fatless A-ZIP/F1 mice have reduced overall marrow cells and HSCs in long bones, yet exhibit an increased number in caudal vertebrae (Zhou et al., 2017). This difference was attributed to the presence of a high number of blood vessels in the tail vertebrae of A-ZIP/F1 mice, a phenomenon not seen in femurs (Zhou et al., 2017). Suppression of marrow vascularization impairs HSC frequency and hematopoietic regeneration (Hooper et al., 2009). In addition to SCF, BMAs also synthesize adiponectin and leptin, which promote HSC proliferation (DiMascio et al., 2007; Poloni et al., 2013). Although the finding that BMA expansion is accompanied by reduced hematopoiesis has conventionally been interpreted to reflect an inhibitory effect of BMAs on hematopoiesis, these data suggest that adipogenesis is an emergency response that produces HSC niche factors and promotes hematopoiesis in most bones. Compared with constructing new perivascular niches, adipogenesis is a faster way to produce HSC niche factors, which involves the promotion of marrow vascularization (Zhou et al., 2017).

To confirm further the connections between BMAs and hematopoiesis in primates, the rhesus macaque model has been used to determine that hematopoietic stem and progenitor cells (HSPCs) reside abreast of BMAs. Furthermore, BMAT-conditioned medium promotes the expansion and differentiation of HSPCs *ex vivo* (Robino et al., 2020). To explore the underlying mechanism, quantitative proteomic examination of BMAT-conditioned medium was performed. A total of 994 proteins were found to be released from BMAT, including TGFB1, FBLN1, IGFBP2, LGALS1, TIMP1, and C3, which have been identified as positive regulators of HSPC differentiation, motility, and adhesion (Robino et al., 2020). Among them, 430 proteins are of microvesicular/exosomal origin, indicating complex composition and paracrine activity. Of note, BMAT contains many types of cells in addition to BMAs, including granulocytes and monocytes/macrophages (Robino et al., 2020). Several proteins identified from BMAT are derived from these cellular neighborhoods of BMAs. Thus, BMAs may also regulate HSPC activity through these immune cells.

The function of BMAs in leukemia is debatable and lineage specific. In acute lymphoblastic leukemia (ALL), *in vitro* and *in vivo* studies indicate that BMAs inhibit T-ALL proliferation (Cahu et al., 2017). In acute myeloid leukemia (AML), Shafat et al. report that AML blasts cocultured with BMA show reduced apoptosis and enhanced proliferation (Shafat et al., 2017). AML blasts promote BMA lipolysis, and the fatty acids generated are transmitted from BMAs to AML cells for β-oxidation. However, a recent study showed that AML caused a reduction in adipocytes in human marrow and AML xenografts (Boyd et al., 2017), suggesting that AML mainly influences the adipocyte population, in addition to promoting the lipolysis of preexisting adipocytes (Boyd et al., 2017). Furthermore, global transcriptome analysis of BMSCs from AML patients or healthy bone marrow donors revealed that adipogenic differentiation is compromised by AML (Boyd et al., 2017). To explore further the relationship between BMA decline and deficient myelo-erythropoiesis in AML, the researchers performed Transwell assays, which showed that BMAs promote myeloid and erythroid lineage maturation. The PPARγ agonist GW1929 was used to stimulate adipogenesis and was found to rescue hematopoietic maturation while suppressing leukemic growth (Boyd et al., 2017). Overall, BMAs promote normal myelo-erythroid maturation and may be a useful therapeutic target to improve bone marrow failure in AML. However, the high sensitivity of BMAs to the change of metabolic status hampers a clear definition of its function in distinct clinical situations and further studies are needed to fully unveil the function of BMA in physiology and different pathology conditions (Zinngrebe et al., 2020).

### BMAs inhibit osteogenesis

Findings in healthy individuals indicate that regardless of age (age 5–88 years), BMAs are negatively related to bone mass (Shen et al., 2007; Shen et al., 2014). BMAs have also been reported to inhibit bone formation and fracture healing, although the underlying mechanism is still under investigation (Ambrosi et al., 2017).

BMA ablation enhances osteogenesis. In a recent study, researchers mated mice bearing diphtheria toxin receptor (DTR), under control of a STOP-flox, to *Adipoq*-Cre mice (Zou et al., 2020). The DTR^*Adipoq*^ mice showed eliminated peripheral and marrow adipocytes following administration of the diphtheria toxin. The bone mass of *Adipoq*-deficiency mice increased 10-fold with 10 days of diphtheria toxin treatment. To exclude the effect of peripheral adipocytes, the authors performed parabiosis between wild-type and DTR^*Adipoq*^ mice and found that diphtheria toxin treatment to control mice induced DTR^*Adipoq*^ mice osteosclerosis, while administration of diphtheria toxin to DTR^*Adipoq*^ mice had no impact on the bone volume of wild-type partners (Zou et al., 2020). Thus, enhanced osteogenesis in DTR^*Adipoq*^ mice is mediated by BMA ablation. To uncover the mechanism of enhanced bone formation following BMA deletion, they further mated DTR^*Adipoq*^ mice to *2.3Col*-GFP reporter mice to characterize osteoblasts. Four days after diphtheria toxin induction, more GFP^+^ cells were observed in DTR^*Adipoq*^ mice. They next mated DTR^*Adipoq*^ *2.3Col*-GFP mice to *TK-3.6Col1a1* mice, a specific strain transduced by mitotic pre-osteoblastic cells (Zou et al., 2020). Diphtheria toxin-induced osteosclerosis is reversed in *TK-3.6Col1a1* mice by ganciclovir, an agent that targets replicating pre-osteoblasts (Zou et al., 2020). These results suggest that BMA ablation promotes pre-osteoblast recruitment and their differentiation into mature osteoblasts. This impact is a result of activation of bone morphogenetic protein receptor (BMPR) and epidermal growth factor receptor pathways. BMAs express chordin-like1 (CHRDL1) and gremlin1 (GREM1), specific BMPR inhibitors, and thus suppress osteogenesis (Zou et al., 2020). Another group reported that BMAs also secrete interleukin (IL)-6 and palmitate to suppress osteoblast activity (Gasparrini et al., 2009; Gunaratnam et al., 2014).

BMAs mediate myeloma-induced osteoblastogenesis suppression. Multiple myeloma is distinguished by overactive bone absorption and impaired bone generation (Palumbo and Anderson, 2011). When BMSCs are cultured with conditioned medium from BMAs obtained from myeloma patients or pre-exposed to myeloma cells, the researchers observed reduced Alizarin red S staining, alkaline phosphatase levels, and osteoblastic gene expression (Liu et al., 2019). Furthermore, using an extramedullary model of osteogenesis and calvarial bone defect, they found that myeloma-associated adipocytes weakened new bone formation (Liu et al., 2019). To explore the mechanism, microarray analysis and qPCR were performed to examine adipokine expression profiles in adipocytes from the bone marrow of patients and healthy controls. Three downregulated genes (adiponectin, adipsin, and visfatin) and one upregulated gene (*Tnfa*) were identified. The changes in expression of these adipokines inhibited osteoblastogenesis (Liu et al., 2019).

BMAs and osteoblasts are derived from the same stem cell, and the direction toward adipocytes occurs at the cost of osteoblast reduction. Thus, regulating the lineage allocation of BMSCs is an effective way to enhance osteoblastogenesis (Suresh et al., 2019; Yu et al., 2019). However, it is notable that the mutual exclusivity between adipocytes and osteoblasts in BM has not yet been proven. During puberty, bone marrow changes from red toward yellow but bone-forming activity has reached its peak (Moore and Dawson, 1990; Devlin and Rosen, 2015). Various animal models show elevated bone mass and excessive BMAT (Ackert-Bicknell et al., 2009).

### BMAs enhance osteoclastogenesis

BMAs promote osteoclastogenesis and recent studies by Hu et al. and Yu et al. have begun to address how BMAs regulate osteoclast formation and bone remodeling (Hu et al., 2021b; Yu et al., 2021). Receptor activator of NF-κB (RANK) and its ligand RANKL play pivotal roles in osteoclastogenesis (Theill et al., 2002). RANKL is predominantly found in osteoblasts, osteocytes, and hypertrophic chondrocytes and binds RANK of osteoclast progenitors to induce osteoclastogenesis (Onji et al., 2021). Notably, BMAs also express RANKL (Fan et al., 2017). Yu et al. crossed *Rankl*-floxed mice with the *Adipoq*-Cre line to uniquely knock out *Rankl* in adipogenic lineage cells (*Adipoq* ^cre^;*Rankl* ^fl/fl^). Under physiological conditions, these mice showed impaired osteoclastogenesis and bone resorption (Yu et al., 2021). In ovariectomy-induced osteoporosis, we found no decrease in cancellous bone density or cortical bone thickness after *Rankl* deletion in adipose cells (Hu et al., 2021b). To explore the role of RANKL in pathological BMA expansion, *Adipoq* ^cre^;*Rankl* ^fl/fl^ mice were administered the PPARγ activator rosiglitazone to increase bone marrow adipogenesis and the possibility of fracture (Aubert et al., 2010). *Adipoq* ^cre^;*Rankl* ^fl/fl^ mice showed similar BMA expansion but a reduced number of osteoclasts compared with control *Rankl* ^fl/fl^ mice (Aubert et al., 2010). These findings suggest that expanded BMA are crucial sources of RANKL for increased osteoclastogenesis and bone resorption. Overall, these two independent studies revealed that BMAs mediate bone remodeling through RANK/RANKL-dependent regulation of osteoclastogenesis in physiological and pathological states. Thus, targeting bone marrow adipogenesis and RANKL signaling in BMAs may be useful to treat osteoporosis.

### BMAs regulate immune function

BMAs contribute to inflammation and plasma cell malfunction inside the bone marrow. Memory T cells and long-lasting plasma cells settle primarily in the marrow to provide protection against recurrent infections (Tokoyoda et al., 2009). To investigate the function of BMAs in immune regulation, global gene expression analysis was performed to compare mRNA expression of adipocytes from human BMAs and white adipose tissue (WAT) (Miggitsch et al., 2019). Several cytokines, including CCL2 CCL5, IL6, IL8, IL10, IL15, CCR7, CCRL2, and CXCL1 were elevated in BMAs, indicating its immune regulatory function within the bone marrow (Miggitsch et al., 2019). Furthermore, the production of reactive oxygen species (ROS) is elevated inside BMAs. ROS secretion accounts for the inhibition of IgG producing plasma cells (Miggitsch et al., 2019).

BMAs promote memory T cell gathering in the bone marrow upon dietary restriction (DR). While WAT collapses after caloric restriction, BMAs are paradoxically increased, with unclear significance. Recent findings have revealed that memory T cells redistribute from the periphery toward the bone marrow in response to the nutritional challenge (Collins et al., 2019). To determine if BMA expansion contributes to memory T cell viability and aggregation, the *Adipoq*-Cre^ERT2^×*Rosa26*-DTA mice were generated to delete BMAs. *Rosa26*-DTA mice carry a loxP-flanked stop cassette linked to the active fragment of diphtheria toxin. When crossed with *Adipoq*-Cre mice, specific ablation of adipocytes is achieved. In mice with reduced BMAs, memory T cells are no longer sustained in the marrow after DR, indicating BMAs are important for memory T cells homing to the bone marrow. Moreover, these T cells show strengthened protection resisting secondary cancers and infections (Collins et al., 2019). Previous studies have revealed that long-chain fatty acids are indispensable for T cell survival, but how BMAs contribute to memory T cell maintenance and homing is still unclear. Collectively, BMAs help maintain and optimize immunological retention upon DR with an unknown mechanism.

## Regulation of bone marrow adipogenesis

An increase in BMAT is a shared reaction to various clinical circumstances and medication, such as diabetes, obesity, anorexia, senescence, and glucocorticoid treatment. Based on the role of BMAs, regulating bone marrow adipogenesis is a promising method for treating bone marrow-related diseases and controlling the differentiation fate of BMSCs is a feasible way.

Several regulators participate in BMSC differentiation. PPARγ, C/EBPα, platelet-derived growth factor receptor β and zinc finger proteins 423, 467, and 521 are well-known factors required for adipogenesis (de Paula and Rosen, 2020); additional regulators are under investigation. Forkhead box P1 (FOXP1) can interact with the CEBPβ/δ complex and RBPjκ to regulate BMSC fate switches (Li et al., 2017). Expressed on BMSCs, Thy-1 (CD90) is a glycosylphosphatidyl-anchored protein of the immunoglobulin family. Thy-1-deficient mice show increased adipogenesis (Picke et al., 2018), and Thy-1 deficiency results in a reduction in Wnt ligand concomitantly with upregulation of the Wnt inhibitors dickkopt-1 and sclerostin, which inhibit osteogenesis (Picke et al., 2018). microRNAs (miRs) participate in cell metabolism by regulating the mRNA degradation of target mRNA. Antagonism of miR-188 can affect the differentiation fate of BMSCs and promote bone formation (Hu et al., 2021a). BMSC differentiation is also epigenetically regulated: histone demethylases KDM4B and KDM6B inhibit adipogenic differentiation of BMSCs *via* elimination of H3K9me3 and H3K27me3 (Ye et al., 2012).

The bone marrow niche also provides information that regulates BMSC lineage commitment. Sensory nerves can induce osteogenic differentiation of BMSCs by downregulating sympathetic nerve activity. Local elevation of prostaglandin E2 triggers EP4 receptors in sensory nerves and inhibits adipogenesis (Hu et al., 2020). Mechanical forces facilitate osteogenic differentiation of BMSCs and prohibit BMA production (Ozcivici et al., 2010). During mechanical loading, modulation of actin regulates ERK and AKT pathways to induce BMSC differentiation (Li et al., 2015). mTORC2 also plays a role in strain-induced cytoskeletal reorganization. Deletion of mTORC2 in BMSCs abolishes osteogenic differentiation and facilitates adipogenic differentiation (Sen et al., 2014). Endocrine molecules also influence BMSC differentiation. Estrogen acts on estrogen receptor-α and has been shown to suppress adipogenesis (Rooney and van der Meulen, 2017). Both BMSCs and BMAs express the follicle-stimulating hormone (FSH) receptor, and inhibition of its interaction with FSH prevents adipogenesis (Liu et al., 2017). Parathyroid hormone (PTH) regulates bone metabolism and inhibits the differentiation of BMSCs toward BMAs (Fan et al., 2017). Moreover, BMAs express PTH1R, and PTH can induce adipogenic lipolysis, which further diminishes adipogenesis in the bone marrow niche (Maridas et al., 2019). Leptin also regulates bone metabolism (Cohen et al., 1996); hypothalamic or subcutaneous administration of leptin has been shown to impair obesity-induced marrow adiposity (Hamrick et al., 2005; Ambati et al., 2010). Overall, several factors control BMSCs differentiation fate, and further work are needed to identify novel regulators.

## Perspective

BMAs are unique adipocytes that reside in the skeletal space. Previous studies have suggested that mature and premature adipocytes exert various influences on hematopoiesis, bone remodeling, and immune regulation in bone marrow niches (Figure 1). However, it is worth noting that the specificity of *Adipoq*-Cre is questionable (Onji et al., 2021). Many studies have targeted BMAs with *Adipoq*-Cre; however, in aged mice, some osteocytes and osteoblasts are also Cre-positive (Mukohira et al., 2019; Yu et al., 2021). Moreover, *Adipoq* is a marker of mature adipocytes as well as their progenitors, MALPs (Yu et al., 2021). Further studies using more specific Cre lines are warranted to uncover the function of BMAs and adipocyte precursors in distinct phases of differentiation.

Lineage allocation of BMSCs is regulated by niche inputs that involve mechanical, neural, and endocrine modulators. Efforts are being made to genetically or pharmacologically manipulate bone marrow adipogenesis, and it may constitute a novel therapeutic strategy for bone marrow-related disorders.
